# Strategies to Achieve High Circularly Polarized Luminescence from Colloidal Organic–Inorganic Hybrid Perovskite Nanocrystals

**DOI:** 10.1021/acsnano.0c03418

**Published:** 2020-07-09

**Authors:** Young-Hoon Kim, Yaxin Zhai, E. Ashley Gaulding, Severin N. Habisreutinger, Taylor Moot, Bryan A. Rosales, Haipeng Lu, Abhijit Hazarika, Roman Brunecky, Lance M. Wheeler, Joseph J. Berry, Matthew C. Beard, Joseph M. Luther

**Affiliations:** † National Renewable Energy Laboratory, Golden, Colorado 80401, United States

**Keywords:** circularly polarized luminescence, formamidinium lead bromide, chiral ligands, colloidal nanocrystals, time-resolved spectroscopy

## Abstract

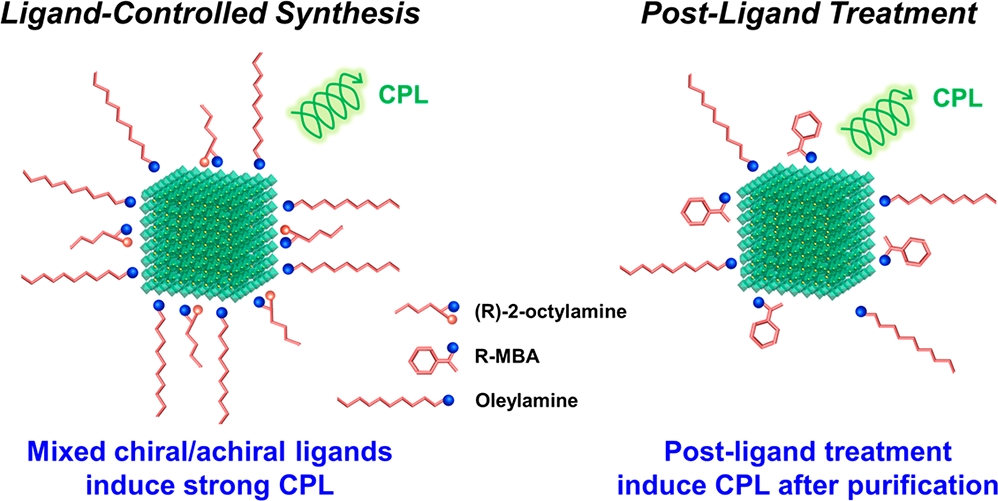

Colloidal metal halide perovskite nanocrystals (NCs) with chiral ligands are outstanding candidates as a circularly polarized luminescence (CPL) light source due to many advantages such as high photoluminescence quantum efficiency, large spin–orbit coupling, and extensive tunability *via* composition and choice of organic ligands. However, achieving pronounced and controllable polarized light emission remains challenging. Here, we develop strategies to achieve high CPL responses from colloidal formamidinium lead bromide (FAPbBr_3_) NCs at room temperature using chiral surface ligands. First, we show that replacing a portion of typical ligands (oleylamine) with short chiral ligands ((*R*)-2-octylamine) during FAPbBr_3_ NC synthesis results in small and monodisperse NCs that yield high CPL with average luminescence dissymmetry *g*-factor, *g*_lum_ = 6.8 × 10^–2^. To the best of our knowledge, this is the highest among reported perovskite materials at room temperature to date and represents around 10-fold improvement over the previously reported colloidal CsPbCl*_*x*_*Br*_*y*_*I_3-*x*-*y*_ NCs. In order to incorporate NCs into any optoelectronic or spintronic application, the NCs necessitate purification, which removes a substantial amount of the chiral ligands and extinguishes the CPL signals. To circumvent this issue, we also developed a postsynthetic ligand treatment using a different chiral ligand, (*R-*/*S*-)methylbenzylammonium bromide, which also induces a CPL with an average *g*_lum_ = ±1.18 × 10^–2^. This postsynthetic method is also amenable for long-range charge transport since methylbenzylammonium is quite compact in relation to other surface ligands. Our demonstrations of high CPL and *g*_lum_ from both as-synthesized and purified perovskite NCs at room temperature suggest a route to demonstrate colloidal NC-based spintronics.

Circularly polarized luminescence (CPL) refers to the differential emission of left- or right-circularly polarized light. CPL can provide information about three-dimensional (*e*.*g*., stereochemical, conformational, chiral) molecular structure in their excited (luminescent) electronic states and therefore be used for a wide variety of technologies including information storage and processing, quantum communication, asymmetric catalysis, 3D displays, agriculture, bioencoding, and photoelectric devices.^1−5^ In order to realize these applications, it is necessary to further develop CPL light sources that produce high luminescence dissymmetry *g*-factors, *g*_lum_ = 2 × (*I*_left_ – *I*_right_)/(*I*_left_ + *I*_right_) (where *I*_left_ and *I*_right_ refer to the PL intensity of left- and right-circularly polarized light, respectively), high photoluminescence quantum efficiency (PLQE), narrow spectral emission, and facile color tunability.^6^

To date, CPL has been achieved by inducing a chiral structural arrangement in emitting materials. The first CPL was reported from chiral lanthanide complexes (*e*.*g*., europium(III), gadolinium(III), and terbium(III)) and chiral actinide complexes (*e*.*g*., sodium uranyl acetate).^3,7^ Lanthanide complexes have demonstrated a high *g*_lum_ of 1 (1.38 in europium(III) complex);^8^ however, the PLQE of these complexes is very low (<40%),^3,7,8^ limiting their use in CPL applications. Chiral organic small molecules exhibiting high PLQE have been developed by attaching enantiomers to phosphorescent and thermal-assisted delayed fluorescent (TADF) molecules; however their *g*_lum_ values have been limited due to the negligible magnetic transition dipole moment (<3 × 10^–3^).^9,10^ Organic molecules are able to achieve an increased *g*_lum_ by self-organizing into helical polymers or supramolecular aggregates and by using aggregation-induced emission; however these strategies reduce the PLQE.^6,11^ Furthermore, organic molecules naturally have broad spectral emission (>50 nm),^12^ limiting their uses in technologies that need high color purity of light, such as 3D displays and bioencoding. Recently, inorganic quantum dots (QDs), such as III–V and II–VI semiconducting QDs and metallic QDs, have been developed for emitting CPL by attaching chiral ligands to their surfaces.^13−16^ This approach could provide a promising path forward to realizing nanostructure-based technologies, but still requires improvements in the *g*_lum_ and PLQE.

Metal halide perovskites (here, perovskites) can fulfill the majority of the requirements of CPL emitting sources (high *g*_lum_, high PLQE, narrow spectral emission, and facile color tunability). A high *g*_lum_ can be achieved due to the potential for high PLQEs and the introduction of chirality in the hybrid structures through (1) the incorporation of chiral organics in the metal halide hybrid framework; (2) attachment of chiral ligands onto the surfaces of metal halide NCs; and (3) the growth of metal halide chiral superstructures.^17−26^Further, metal halide perovskites have narrow spectral emission (full width at half-maximum (fwhm) ≈ 20 nm), allowing for ultrahigh color purity (color gamut >95% in International Telecommunication Union Recommendation BT 2020 (Rec. 2020) standard) and facile and wide color tunability (400 nm < λ < 800 nm).^12,27^ CPL from layered metal halide systems has been demonstrated by incorporating chiral organic molecules as the A-site cation in polycrystalline bulk films. For example, chiral *R*-,*S*-methylbenzylammonium (*R*-,*S*-MBA) was incorporated as an A-site cation in layered two-dimensional (2D) single crystals ((*R*-,*S*-MBA)_2_PbI_4_) and achieved CPL at 77 K.^22^ However, as the temperature increases, the CPL distinctly decreases, and the crystals even do not exhibit any PL at room temperature due to significant nonradiative recombination.^28^ Chiral quasi-2D (“Ruddlesden–Popper”) metal halide polycrystalline bulk films that incorporate chiral A-site cations ((*R*-,*S*-MBA)_2_(MA)_*n*−1_Pb*_n_*Br_3*n*–1_ with *n* = 2) were reported to maintain high PLQE (90%) at room temperature, but did not show any CPL at room temperature.^24^ Lack of performance at room temperature is a significant limitation for their potential implementation into CPL-based applications.

Colloidal perovskite nanocrystals (NCs) offer a route for addressing the shortcomings of other CPL emitting systems because perovskite NCs have inherently high PLQE at room temperature, and their native organic surface ligands offer an opportunity to achieve CPL from the perovskite crystals.^29,30^ Attempts to induce CPL from CsPbCl*_*x*_*Br*_*y*_*I_3-*x*-*y*_ NCs include direct synthesis using chiral α-octylamine as a surface ligand^31^ and assembly of nonchiral NCs using a chiral organogel.^32^ These approaches exhibit limited *g*_lum_ < 7 × 10^–3^.^33^ Organic–inorganic hybrid perovskite (OIP) NCs employing an organic A-site cation (*e*.*g*., formamidinium (FA))^34−36^ can have higher *g*_lum_ because the use of OIP NCs offers a wide selection of methods to induce chirality, including by incorporating chiral organic molecules at the A-site cation^20,22,23^ and/or as the surface ligand.^37^ However, there have been no reports of CPL from OIP NCs to date.

Beyond synthesis, incorporation of these NCs into films for use in various optoelectronic applications such as light-emitting diodes or photovoltaics requires that the NCs are purified after synthesis.^38−40^ This purification step tends to remove ligands from the NC’s surface, which can diminish their CPL. Therefore, previously reported NCs synthesized with chiral ligands^31^ and NCs assembled using chiral organogels^32^ do not maintain CPL after being processed into films and, thus, have limitations for use in spintronic applications. Therefore, it is critical to develop a method that maintains or reestablishes the CPL from the OIP NCs after the necessary NC purification processes.

Here, we report two simple but effective strategies to achieve high CPL responses from colloidal FA-lead bromide (FAPbBr_3_) NCs at room temperature with chiral surface ligands (Figure 1). First, we substitute a portion of typical ligands (oleylamine (OAm), which binds as oleylammonium (OAmH^+^) to the NC surfaces)^39^ with short chiral ligands ((*R*)-2-octylamine) during the synthesis of the FAPbBr_3_ NCs. This leads to small and relatively monodisperse NCs exhibiting high CPL with a *g*_lum_ of 6.8 × 10^–2^. To the best of our knowledge, this is the highest yet reported for colloidal perovskite NCs and represents around a 10-fold improvement over the previously reported CsPbCl*_*x*_*Br*_*y*_*I_3-*x*-*y*_ NCs.^31,32^ However, we find that postsynthetic purification (standard NC washing procedure with methyl acetate (MeOAc)),^38^ which is necessary for use of perovskite NCs in optoelectronic or spintronic devices, removes the chiral ligands and extinguishes the CPL response. Therefore, we develop a postsynthetic ligand treatment employing chiral (*R*-/*S*-) methylbenzylammonium bromide (*R*-,*S*-MBA:Br) to recover left- and right-handed CPL with an average *g*_lum_ = ±1.18 × 10^–2^ from the purified NCs, which is again higher than what has been previously reported for all-inorganic perovskite NCs.^31,32^

**Figure 1:**
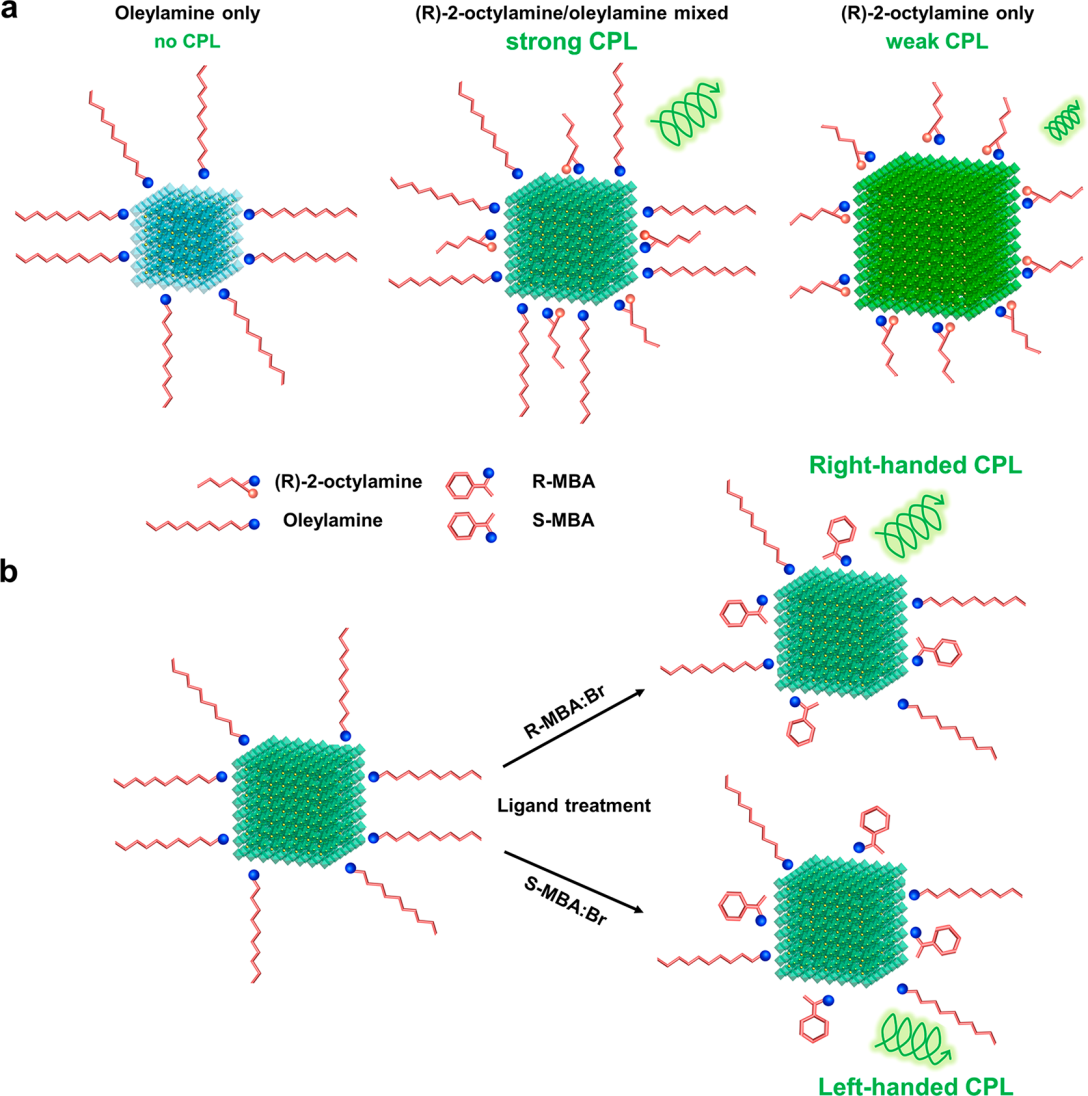
Schematic illustrations of (a) ligand-controlled synthesis of FAPbBr_3_ NCs with (*R*)-2-octylamine and (b) postsynthetic ligand treatment of FAPbBr_3_ NCs with *R*-,*S*-MBA:Br (note: for simplicity, oleic acid is not illustrated in the depiction).

## Results and Discussion

We employ a hot-injection method for the synthesis of size-tunable FAPbBr_3_ NCs.^29^ To directly introduce chirality into NCs during synthesis, we utilize (*R*)-2-octylamine as a chiral ligand due to its structural similarity with OAm and ability to dissolve PbBr_2_ precursor, unlike *R*-,*S*-methylbenzylamine,^20,22,23^ which cannot because the phenyl group is too large to penetrate the PbBr_2_ stacks.^41,42^ The synthesis is conducted at 80 °C because FAPbBr_3_ NCs with pure (*R*)-2-octylamine (without OAm) have low thermal stability arising from the short hydrocarbon chain in (*R*)-2-octylamine^43^ and thus cannot be synthesized at high temperatures typically used for perovskite NCs (160–180 °C).^39,44^ We control the ratio of (*R*)-2-octylamine to OAm during synthesis at a constant reaction temperature of 80 °C while maintaining the same concentration of oleic acid (OA) and analyze photophysical and chiroptical properties of as-synthesized FAPbBr_3_ NCs.

**Figure 2:**
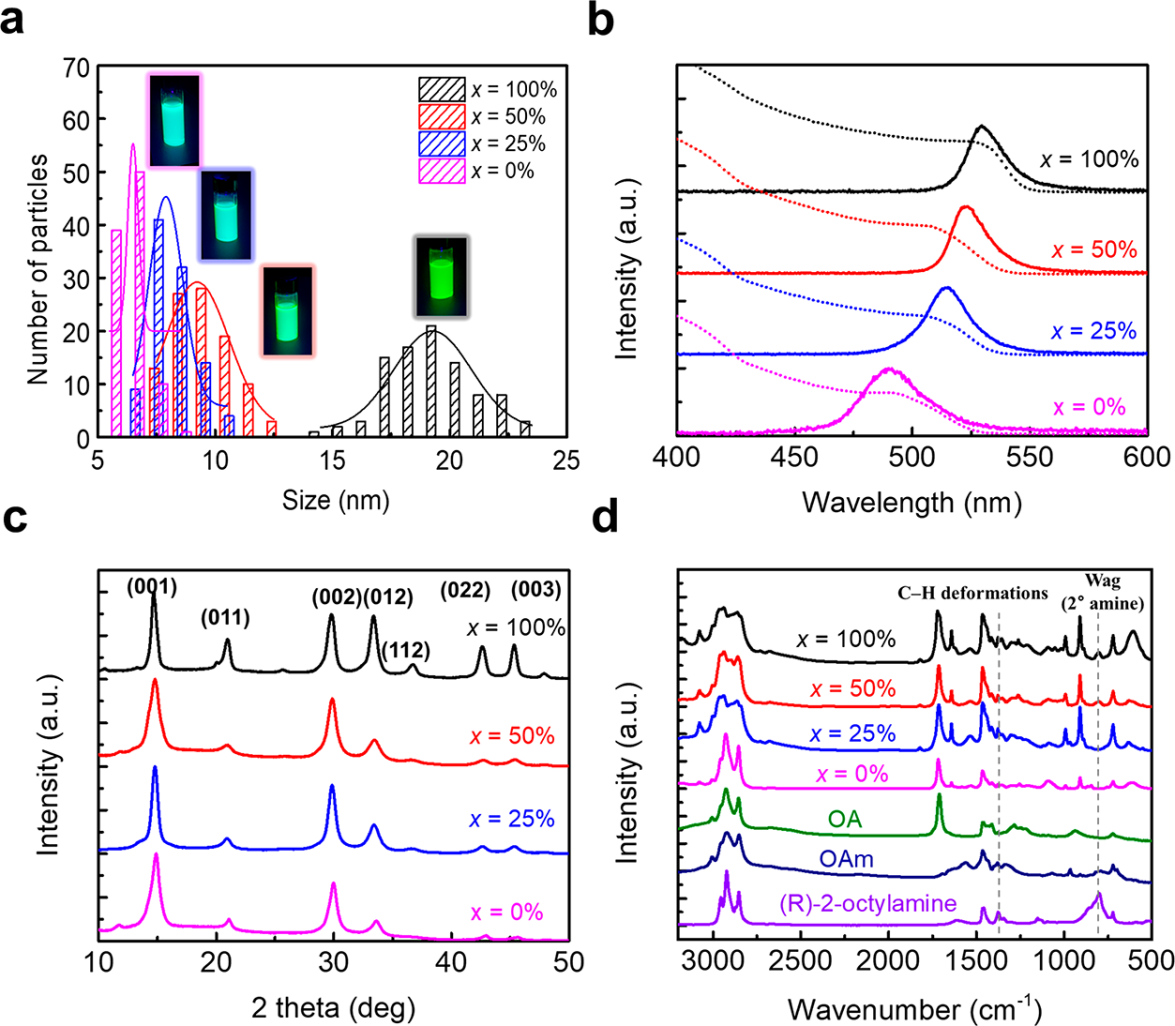
(a) Size distribution histograms and photographs under a λ = 350 nm Xe lamp (inset), (b) PL (line) and absorption (dot) spectra, (c) XRD patterns of FAPbBr_3_ NCs with different (*R*)-2-octylamine concentration *x*. (d) FTIR spectra of oleic acid, oleylamine, (*R*)-2-octylamine, and FAPbBr_3_ NCs with different *x*.

As (*R*)-2-octylamine concentration *x* decreases from 100% (pure (*R*)-2-octylamine) to 0% (pure OAm) at a constant reaction temperature of 80 °C, the size of the FAPbBr_3_ NCs gradually decreases from ∼19 nm to ∼7 nm, as measured by transmission electron microscopy (TEM) (Figures 2a, S1). We attribute this decrease in size to the fact that long-chain ligands (OAm) have higher steric hindrance than short-chain ligands ((*R*)-2-octylamine) and, therefore, prevent diffusion of precursor species to the surface of NCs,^45−47^ impeding NC growth. The decrease in NC size with decreasing *x* is confirmed by both PL and absorption spectra. As *x* decreases from 100% to 0%, the PL spectrum gradually shifts toward higher energies (shorter wavelengths) from 528 nm to 490 nm due to quantum confinement, as does the absorption spectra (Figure 2b). The PL spectra also broaden, *e*.*g*., fwhm ≈ 18 nm for NCs with *x* = 100% to ∼32 nm for NCs with *x* = 0% due to increased size-dependent bandgap variation from quantum confinement when the NCs are smaller.^30^ FAPbBr_3_ NCs also exhibit blue-shifted emission under UV illumination as *x* decreases (insets of Figure 2a).

All FAPbBr_3_ NCs show clear X-ray diffraction (XRD) patterns with prominent (001), (002), and (012) peaks at ∼14.69°, ∼29.77°, and ∼33.39° (Figure 2c), which is consistent with a previous report.^48^ As (*R*)-2-octylamine concentration *x* decreases, the diffraction peaks shift to a higher 2θ angle, which indicates a decrease in the lattice parameter from 5.99 Å (for *x* = 100%) to 5.95 Å (for *x* = 0%) (Figure S2a,b), possibly due to an increase in surface tension as the NC size decreases.^49−51^

Because CPL is affected by the chiral ligands attached to the NC surfaces, we conducted Fourier transform infrared (FTIR) spectroscopy to study the surface chemistry of FAPbBr_3_ NCs (Figure 2d). We confirm the presence OA by ν(C=O) = 1720 cm^–1^ and ν_s_(COO^–^) = 1412 cm^–1^, and both OA and OAm by ν(C–H*_*x*_*) (2850–3000 cm^–1^), ν(C=C–H) = 3005 cm^–1^, and ν(C–H_2_) = 1466 cm^–1^ where ν is stretching and ν_s_ is the symmetric stretching mode. Similarly, (*R*)-2-octylamine is identified by the stretches of its secondary (2°) amine group. NCs with (*R*)-2-octylamine (*x* = 100, 50, 25%) show a C–H deformation peak at 1371 cm^–1^ and an N–H wagging peak of 2° amines at 800 cm^–1^, confirming that (*R*)-2-octylamine is attached to the NC surfaces.^52^ Furthermore, NCs with (*R*)-2-octylamine (*x* = 100, 50, 25%) show broadened FTIR peaks at 2850–3000 cm^–1^, which typically arise when there are multiple local chemical environments.^53^ Therefore, we attribute the broadened FTIR peaks observed in the NCs with (*R*)-2-octylamine to a chemical environment caused by the 2° amine in (*R*)-2-octylamine (*e*.*g*., bonding of the 2° ammonium in (*R*)-2-octylamine with COO^–^ or Br^–^ on the NC surface or proton exchange with primary (1°) ammonium (FA^+^, NH_3_^+^ in FAPbBr_3_ crystals or OAmH^+^)). The combination of PL, XRD, and FTIR indicates that the use of (*R*)-2-octylamine in this study affects the size and surface chemistry of FAPbBr_3_ NCs during synthesis; however in future work, one could potentially use the chiral ligands to manipulate the growth of NCs in advantageous ways.

**Figure 3:**
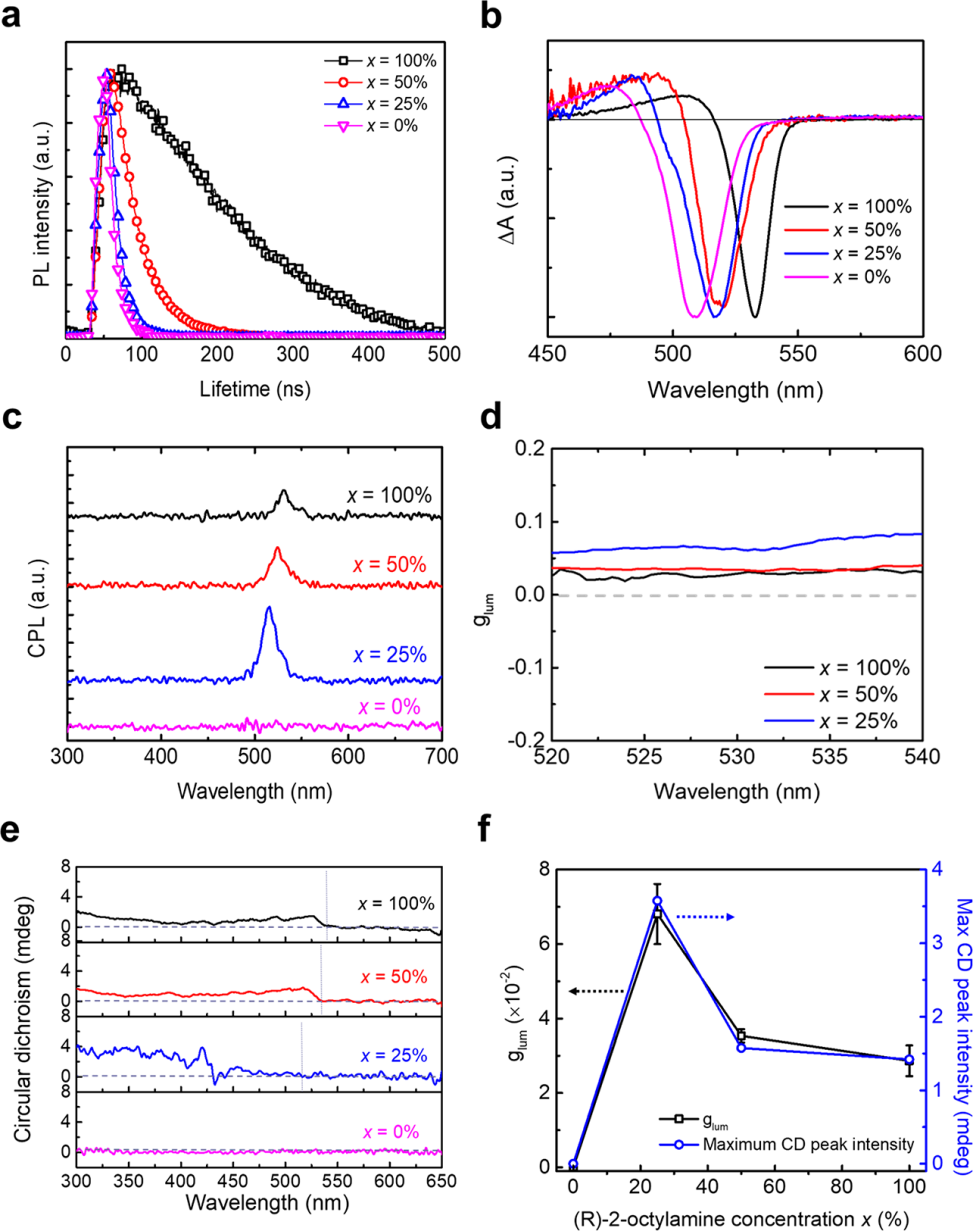
(a) Time-resolved photoluminescence (TRPL) decay kinetics, (b) normalized transient absorption (TA) spectra at 2 ps, (c) circular polarized luminescence (CPL), (d) *g*_lum_, (e) circular dichroism (CD) spectrum, and (f) average *g*_lum_ values measured at 520 ≤ λ ≤ 540 nm and maximum CD peak intensity in FAPbBr_3_ NC solutions with different (*R*)-2-octylamine concentration *x*.

Since CPL response is governed by radiative recombination within NCs and the interplay that chiral ligands impart on the recombination dynamics, we conducted time-resolved PL (TRPL) to understand exciton recombination dynamics in the FAPbBr_3_ NCs (Figure 3a). With a decrease in (*R*)-2-octylamine concentration *x*, the resulting FAPbBr_3_ NCs show a gradually decreasing PL lifetime from 231 ns at *x* = 100% to 12.2 ns at *x* = 0% due to increased exciton confinement in the smaller NCs.^30,54^Figure 3b shows transient absorption (TA) spectra measured at a delay time of 2 ps. The spectra are normalized at the ground state bleaching peaks to show the trend. We observe a bleach of the exciton, which originates from state-filling in all the samples, and blue-shifts as *x* decreases (Figure S3), which are consistent with absorption and PL spectra and further confirm a decrease of NC size (Figure 2b). FAPbBr_3_ NCs with 25% ≤ *x* ≤ 100% exhibit similar PLQEs of 57–60%, but NCs with *x* = 0% give a reduced PLQE of 32.6% due to increased nonradiative recombination at surface defects which arise from the increased surface/volume ratio in small NCs.

To achieve CPL response from FAPbBr_3_ NCs, we have incorporated chiral ligands during synthesis and demonstrated the effects of chiral ligands on size, surface chemistry, and photophysics of NCs. Now, we will discuss the CPL properties of FAPbBr_3_ NCs as a function of (*R*)-2-octylamine concentration *x*. As *x* decreases from 100% to 25%, FAPbBr_3_ NCs show blue-shifted and intensified CPL and increased *g*_lum_ from 2.8 × 10^–2^ to 6.8 × 10^–2^, measured at 520 ≤ λ ≤ 540 nm (Figure 3c,d), which is the highest value reported to date for perovskite materials at room temperature. We also measured circular dichroism (CD) of synthesized FAPbBr_3_ NCs. FAPbBr_3_ NCs with chiral ligands have CD spectra onsets at 500–540 nm (Figure 3e), which are markedly different from that of (*R*)-2-octylamine (onset at 293 nm) (Figure S4); this confirms that chiral surface ligands induce optical chirality into the inorganic Pb–Br framework, and CPL and CD signals of NCs do indeed originate from FAPbBr_3_ crystals. As *x* decreases, NCs show blue-shifted CD spectra and increased maximum CD peak intensity (from 1.46 mdeg for NCs with *x* = 100% to 3.53 mdeg for NCs with *x* = 25%); these results corroborate the results of high *g*_lum_ in small NCs with a low concentration of chiral ligands (Figure 3f). FAPbBr_3_ NCs without (*R*)-2-octylamine (*x* = 0%) do not show any CPL or CD signal as expected due to the absence of chiral ligands.

Although one may expect that NCs with the highest concentration of injected chiral ligands during synthesis would yield the largest CPL intensity, we instead find that the NCs with a relatively low concentration of injected chiral ligands have the largest CPL. This demonstrates that the absolute number of injected chiral ligands is not the only important factor for determining chiroptical properties in NCs. Kuznetsova *etal*.^55^ also reported that a low concentration of chiral ligands (cysteine) induces the highest chiroptical properties on CdSe/CdS QDs, which is due to different binding configurations of ligands on the QD surface at different ligand concentrations. Additionally, Moshe *etal*.^56^ and Yao *etal.*(57) reported that smaller QDs induce a larger electronic coupling between the surface chiral ligands and the more confined charge carrier wave functions. In our experiments, (*R*)-2-octylamine has one functional group which can be coordinated to the perovskite crystals. Therefore, we attribute the high CPL of FAPbBr_3_ NCs with a relatively low concentration of injected chiral ligands during synthesis to the fact that small NCs (1) have a high surface-to-volume ratio where more surface chiral ligands can attach to the surface and (2) have a minimized distance between surface chiral ligands and electronic states in the core NCs, inducing larger effects of chiral ligands (*i*.*e*., electronic coupling,^56,57^ chiral ligand-induced surface lattice distortion,^31^ or surface defects^58^) on the electron–hole wave functions inside NCs. Further detailed research is needed to determine the exact mechanism of chirality transfer in the OIP NCs.

In order to use NCs in any optoelectronic device, the NCs must form a high-quality thin film, which necessitates purification of the NCs prior to their deposition.^38−40^ However, the common purification method using MeOAc^38^ removes the chiral ligands from the as-synthesized NC surfaces and extinguishes the CPL response (Figure S5a). The removal of (*R*)-2-octylamine is confirmed by a sharpening of the FTIR peaks at 2850–3000 cm^–1^ and disappearances of the C–H deformation peak at 1371 cm^–1^ and of the N–H wagging peak in the 2° amine at 800 cm^–1^ (Figure S5b). To reactivate a CPL response, we performed postsynthetic ligand treatment to the purified NCs by mixing a dispersion of purified NCs with a saturated solution of the chiral ligand in ethyl acetate (EtOAc). Here, we chose *R*-,*S*-MBA:Br as the chiral ligand because it is easily characterized spectroscopically by its phenyl group when attached to the NC surfaces. We chose EtOAc as the solvent because EtOAc preserves the crystal structure of the perovskite NCs while being able to dissolve chiral ligands of interest.^38−40^

**Figure 4:**
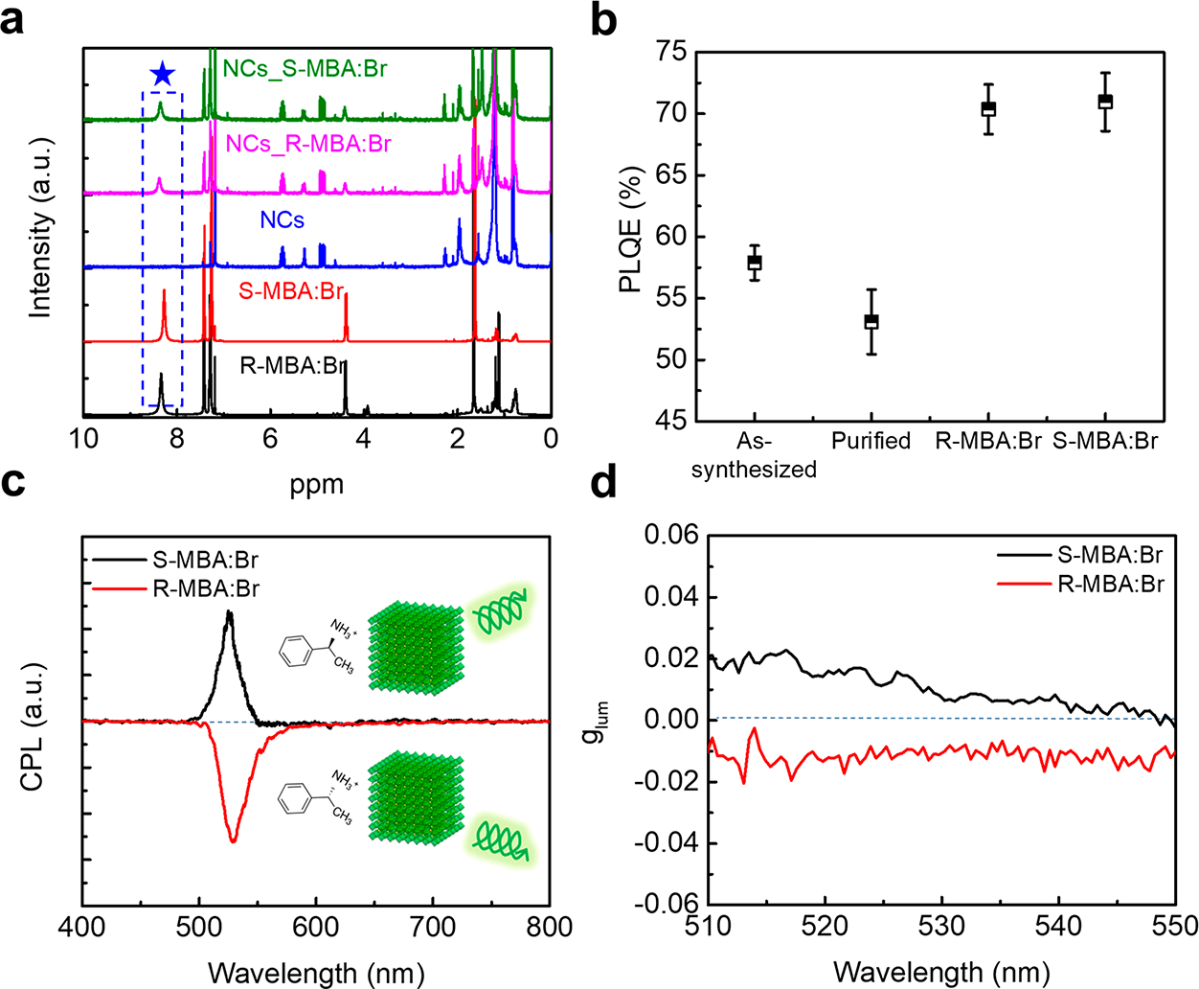
(a) ^1^H NMR spectra of *R*-,*S*-MBA:Br, purified FAPbBr_3_ NCs, and ligand-treated FAPbBr_3_ NCs with *R*-,*S*-MBA:Br, (b) PLQE of as-synthesized FAPbBr_3_ NCs, purified NCs, and ligand-treated NCs with *R*-,*S*-MBA:Br, and (c) CPL and (d) *g*_lum_ of ligand-treated FAPbBr_3_ NCs with *R*-,*S*-MBA:Br.

We conducted ^1^H NMR to verify that *R*-,*S*-MBA:Br was attached to the NC surfaces after the postsynthetic ligand treatment (Figure 4a). The purified FAPbBr_3_ NCs show peaks at chemical shift (δ) ∼0.8 ppm, ∼1.2 ppm, and ∼1.9 ppm, which arise from an alkyl proton, indicating that some OA and OAm ligands remain on the NC surfaces after purification.^39^ The *R*-,*S*-MBA:Br ligands exhibit a sharp NMR peak at δ ∼8.3 ppm, which corresponds to aromatic protons and does not overlap with any peaks from the purified NCs. Postsynthetic ligand treated NCs with *R*-,*S*-MBA:Br show an aromatic-proton NMR peak that is broadened and shifted downfield to 8.35–8.4 ppm compared to that of pure *R*-,*S*-MBA:Br (Figure S6). These changes indicate *R*-,*S*-MBA:Br molecules associate with the NC surface.^59^ FTIR spectra show a relative decrease in the peak intensity of the ν(C–H_2_) and ν(C=O) after ligand treatment (Figure S7), indicating that some *R*-,*S*-MBA:Br ligands are attached to the NC surfaces by replacing either oleylammonium (from OAm) or oleate (from OA).

Having demonstrated chiral ligands attach to the purified NC surfaces through the postsynthetic ligand treatment, we next investigate whether they affect exciton recombination dynamics and confer chiral emission in NCs. Purified NCs exhibit a PLQE of ∼53% but upon postsynthetic ligand treatment, the PLQE rises significantly to ∼70.4% for NCs with *R*-MBA:Br and to ∼71.0% for NCs with *S*-MBA:Br (Figure 4b). TRPL maps show that the PL lifetime decreases after purification but is then rectified after the ligand treatment (Figure S8). These results indicate that *R*-,*S*-MBA:Br passivates the surface defects induced by detached ligands from the NC surface during the purification and thus reduces nonradiative recombination. Indeed, the treatment of the purified NCs with *R*-,*S*-MBA:Br ligands induces CPL responses with average *g*_lum_ = ±1.18 × 10^–2^ at 510 ≤ λ ≤ 550 nm at room temperature (Figure 4c,d). The success of this postsynthetic ligand treatment method is of critical importance because it solves two problems with the current methods for inducing CPL on NCs: chiral ligands are often removed during the purification process leading to extinguishment of CPL and long native ligands (OA, OAm) often hamper charge transport, both of which are necessary for implementation of NCs into spintronic-based applications.

Here, we suggest some future research directions to further increase the *g*_lum_ of perovskite NCs. Basically, dynamic ligand binding to the NC surface and steric hindrance of chiral ligands arising from the bulky alkyl group limit their strong and compact attachment to the NC surface. Therefore, we expect that other chiral ligands (1) with different functional groups (*e*.*g*., carboxylic acid, phosphonic acid) that adhere more strongly to the NC surface and (2) with short aliphatic groups that have less steric hindrance and can attach to the NC surface more compactly can boost the *g*_lum_ of perovskite NCs. We also suggest that chiral zwitterionic ligands that have both acid (*e*.*g*., SO_3_^–^, COO^–^, and PO_3_^–^) and amine (NH_3_^+^) groups can be effective strategies to realize efficient chiral NCs because zwitterionic ligands can exhibit various binding modes on the NC surfaces such as bidentate and tridentate.^55^

## Conclusions

In conclusion, we report two strategies to achieve a high CPL response from colloidal FAPbBr_3_ NCs at room temperature by exploiting chiral surface ligands either during the synthesis or by a postsynthetic ligand treatment. For the ligand-controlled synthesis, we provide insight into the effects of the chiral ligand concentration on the size, surface chemistry, and photophysics on colloidal FAPbBr_3_ NCs. A chiral (*R*)-2-octylamine concentration of 25% was shown to maximize the CPL and achieved an average *g*_lum_ of 6.8 × 10^–2^. To the best of our knowledge, this is the highest value yet reported for perovskites at room temperature and represents around 10-fold improvement over the previously reported colloidal CsPbCl*_*x*_*Br*_*y*_*I_3-*x*-*y*_ NCs. We also develop and implement a postsynthetic ligand treatment process that attaches chiral *R*-,*S*-MBA:Br on the surface of purified NCs, inducing CPL responses with an average *g*_lum_ of ±1.18 × 10^–2^. This postsynthetic method enhances device applicability for these systems. Taken together, we have illustrated how chiral ligands can suppress the nonradiative recombination by passivating defects on the NC surfaces, thus increasing the PLQE. Incorporating chiral organic molecules into OIP NCs provides a direct interaction between the ligand and the emitter, which is not possible using core–shell structures. Our demonstrations of high CPL and *g*_lum_ from both as-synthesized and purified OIP NCs at room temperature, in conjunction with high PLQE, establish routes to induce chiroptical properties in perovskite NCs and provide a way to devise spintronics based on colloidal NCs.

## Methods and Experimental Details

### Chemicals

Formamidinium acetate (FA-acetate, 99%), oleic acid (OA, technical grade 90%), oleylamine (OAm, technical grade 70%), 1-octadecene (ODE, technical grade 90%), hexane (reagent grade ≥95%), octane (anhydrous, ≥99%), methyl acetate (MeOAc, anhydrous 99.5%), ethyl acetate (EtOAc, anhydrous 99.5%), *R*-methybenzylamine (98% purity), *S*-methybenzylamine (98% purity), hydrobromic acid (ACS reagent, 48%), and ethyl acetate (EtOAc, anhydrous, 99.8%) were purchased from Sigma-Aldrich. (*R*)-2-Octylamine (98% purity) was purchased from Alfa Aesar.

### Synthesis of FA-Oleate Precursor

In a three-necked round-bottom flask, 15 mmol (1.563 g) of FA-acetate, 15 mL of OA, and 15 mL of ODE were degassed under a vacuum at room temperature and 50 °C for 30 min, respectively. The temperature was then increased to 120 °C under N_2_ and kept at this temperature.

### Synthesis of FAPbBr_3_ NCs

In a three-necked round-bottom flask, 0.74 mmol (0.272 g) of PbBr_2_ and 25 mL of 1-ODE were degassed under vacuum at room temperature and 120 °C for 30 min, respectively. Mixtures of 12.1 mmol (4 mL) of OA and 6.05 mmol of amine ligands with different ratios of (*R*)-2-octylamine to OAm (1:0, 0.5:0.5, 0.25:0.75, 0:1) were preheated to 120 °C, then injected into the PbBr_2_ mixture under vacuum. After PbBr_2_ was fully dissolved, the solution became clear. Then the temperature of the solution was reduced to 80 °C to prevent rapid growth of FAPbBr_3_ crystals. Under N_2_ flow, 5 mL of the FA-oleate stock solution was swiftly injected into the PbBr_2_ mixture, which was then quenched by immersing the flask in an ice bath. After cooling to room temperature, the solution was centrifuged at 7500 rpm for 5 min to remove unreacted ligands and 1-ODE. The precipitate was dispersed in 5 mL of hexane, then centrifuged at 7500 rpm for 5 min to remove the precipitated large particles. The supernatant NC solution was stored in a refrigerator.

### Synthesis of *R*-,*S*-MBA:Br

In a one-necked round-bottom flask in an ice bath, 78 mmol (10 mL) of *R*-,*S*-MBA and 30 mL of ethanol were added; then 116 mmol (13.2 mL) of HBr aqueous solution was added dropwise into the flask under vigorous stirring. The solution was kept stirring in an ice bath overnight. The yellowish precipitate was collected by evaporation of solvents at 70 °C for 30 min, then washed thoroughly by repeated redissolution in ethanol and recrystallization in diethyl ether until colorless. The white precipitate was dissolved in a small amount of hot ethanol to make a saturated solution, then recrystallized in a freezer; a temperature drop reduced the saturation concentration of *R*-,*S*-MBA:Br in ethanol and induced recrystallization. The recrystallized precipitate was dried under vacuum overnight.

### Purification and Ligand Treatment of FAPbBr_3_ NCs

To purify the FAPbBr_3_ NCs, 10 mL of MeOAc was added to 5 mL of a stock solution; then it was centrifuged at 7500 rpm for 5 min. The resulting precipitate was redispersed in 5 mL of hexane and centrifuged again at 7500 rpm for 5 min to discard the aggregated particles. To conduct the ligand treatment of FAPbBr_3_ NCs, a saturated solution of *R*-,*S*-MBA:Br in EtOAc was prepared by sonicating 200 mg of *R*-,*S*-MBA:Br in 20 mL of EtOAc, then centrifuging at 3500 rpm for 5 min. The 1 mL of saturated *R*-,*S*-MBA:Br solution was mixed with 5 mL of purified FAPbBr_3_ NCs; the mixture was stirred mildly for 5 min at room temperature and then centrifuged at 7500 rpm for 5 min to precipitate the aggregated particles; the supernatant was collected.

### Transmission Electron Microscopy Measurement

FAPbBr_3_ NC solutions were dropped on the carbon-coated copper mesh grids (CF200-Cu, Electron Microscopy Sciences). The transmission electron microscopy experiment was performed using a FEI Tecnai ST30 TEM operating at an acceleration voltage of 300 keV.

### ^1^H NMR Spectroscopy

NMR were taken on a Bruker 400 Avance III NMR using a standard proton pulse (zg), 64 scans, 4.0 s collection times, and a 25.0 s delay between scans at 25 °C.

### Time-Resolved photoluminescence

The samples were excited at 450 nm at a low fluence (≪10^15^ s^–1^) using a supercontinuum fiber laser (NKT Photonics, Super K) operating at 5 MHz as the excitation source. The emission was collected with a Hamamatsu C10910-04 streak camera.

### Photoluminescence Quantum Efficiency

PLQEs of FAPbBr_3_ NC solutions were measured using a 100 mm integrating sphere (Labsphere) integrated with the spectrometer (SpectraPro HRS 500, Princeton Instruments). FAPbBr_3_ NC solutions were excited using a 450 nm Xe lamp.

### Circularly Polarized Luminescence

FAPbBr_3_ NCs were excited by a linearly polarized laser at 405 nm (Figure S9). The PL emission was separated into σ^+^ and σ^–^ by rotating a broadband quarter-wave plate (400–800 nm, Thorlabs) followed by a linear polarizer. The polarized emission was collected in free space and measured using a spectrometer (MAYA 2000pro, OceanOptics). The experimental apparatus was calibrated with a depolarized light source, from which we obtained a systematic error of <0.2% in measuring the circular light polarization.

### Transient Absorption

The transient reflection measurement is based on the Ti:sapphire laser amplifier (Continuum Integra, 800 nm, pulse duration ∼100 fs, ∼3 mJ/pulse, and 1 kHz repetition rate) and the pump–probe transient reflection spectrometer (Helios, Ultrafast System). The fundamental laser pulse is generated by a Ti:sapphire amplifier and then split into two parts by a beam splitter. One beam is sent to an optical parametric amplifier to generate the pump pulse with tunable wavelength, and its intensity is attenuated by two neutral density filter wheels. The other part of the fundamental pulse is focused into a sapphire crystal to generate a white-light continuum (450–800 nm), which is used as the probe. The probe pulses are delayed in time with respect to the pump pulses using a motorized translation stage mounted with a retroreflecting mirror. The pump and probe are spatially overlapped on the surface of the sample. Both the pump and probe beam are normally incident on the sample. The size of the focused spot at the sample position for the probe and pump beams is around 200 and 600 μm, respectively.

### Fourier-Transform Infrared Spectroscopy

FTIR measurements were done in an Ar glovebox on a Bruker Alpha FTIR spectrometer using a diffuse reflectance infrared Fourier transform spectrometer attachment with a resolution of 4 cm^–1^. Background measurements were taken on blank substrates, and subsequent sample measurements were taken as an average of 24 scans. Spectra were baseline-corrected using the concave rubberband correction method. Background measurements were taken on air, and subsequent sample measurements were taken as an average of 24 scans.

### Circular Dichroism Spectroscopy

CD measurements were carried out using a Jasco J-715 spectropolarimeter with the samples as a liquid suspension in a 1 mm path length quartz cuvette. The spectra obtained were single scans. The CD spectra of different constructs was monitored from 300 to 650 nm.

## Supporting Information Available

Additional data containing TEM, XRD, transient absorption spectroscopy, and CD spectra of as-synthesized FAPbBr_3_ NCs, and CPL, NMR, and FTIR of ligand-treated FAPbBr_3_ NCs (PDF)
