# Supplementary material for: Strategies to Achieve High Circularly Polarized Luminescence from Colloidal Organic–Inorganic Hybrid Perovskite Nanocrystals
Source: ACS Nano. 2020 Jul 9;14(7):8816–25. doi: 10.1021/acsnano.0c03418 (PMC10906077; doi:10.1021/acsnano.0c03418)
Supplement: Supplementary file 1 — nn0c03418_si_001.pdf [file nn0c03418_si_001.pdf]

Supporting Information for

Strategies to Achieve High Circularly Polarized Luminescence from Colloidal Organic-Inorganic Hybrid Perovskite Nanocrystals

Young-Hoon Kim,† Yaxin Zhai,† E. Ashley Gaulding,† Severin N. Habisreutinger,† Taylor Moot,† Bryan A. Rosales,† Haipeng Lu,† Abhijit Hazarika,† Roman Brunecky,† Lance M. Wheeler,†

*Joseph J. Berry,† Matthew C. Beard,† Joseph M. Luther† **

† National Renewable Energy Laboratory, Golden, Colorado 80401, United States

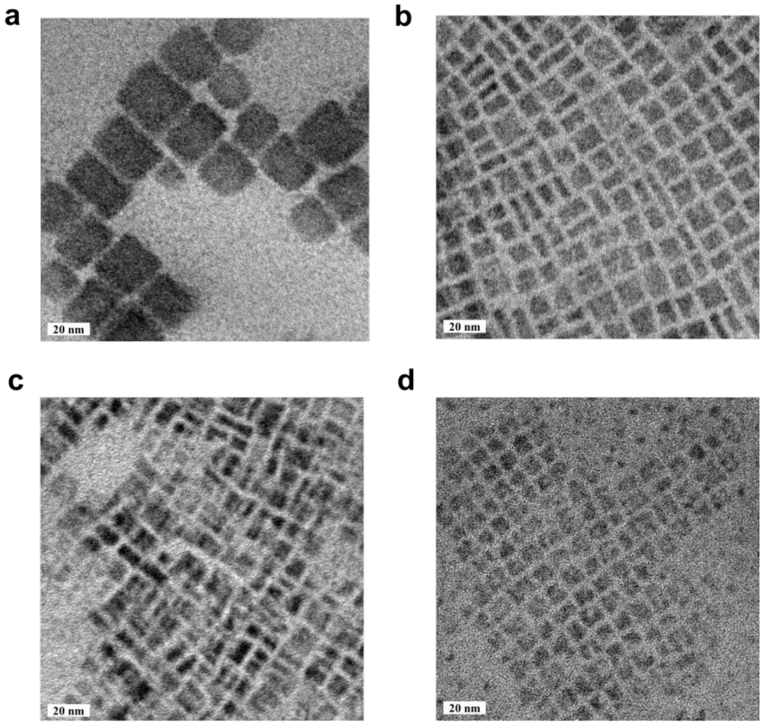

Figure S1. TEM images of $FAPbBr_3$ NCs with different (R)-2-octylamine concentration *x*; a) *x* = 100%, b) *x* = 50%, c) *x* = 25%, d) *x* = 0%.

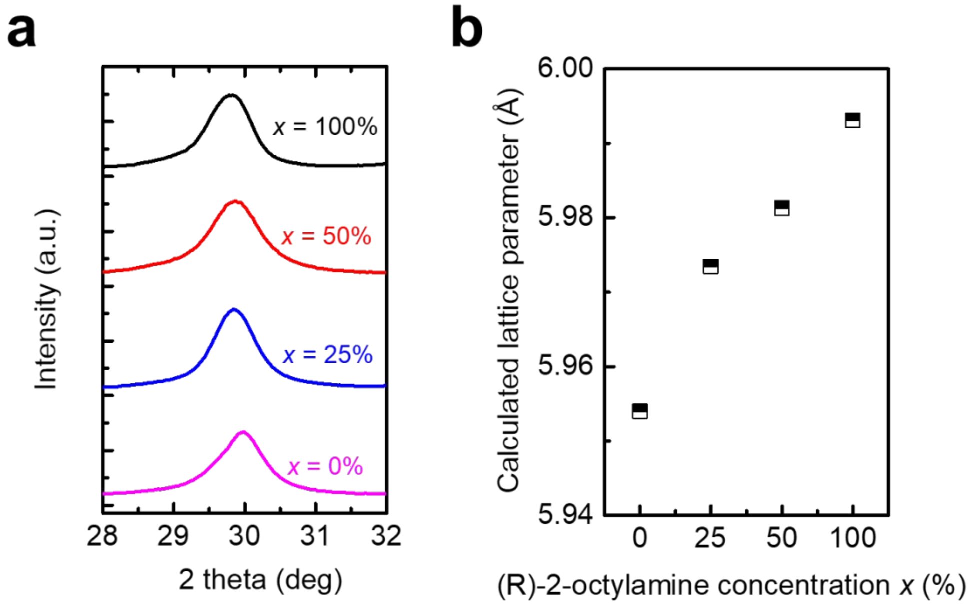

Figure S2. a) Magnified XRD patterns (28-32 deg) and b) calculated lattice parameter of $FAPbBr_3$ NCs with different (R)-2-octylamine concentration *x*.

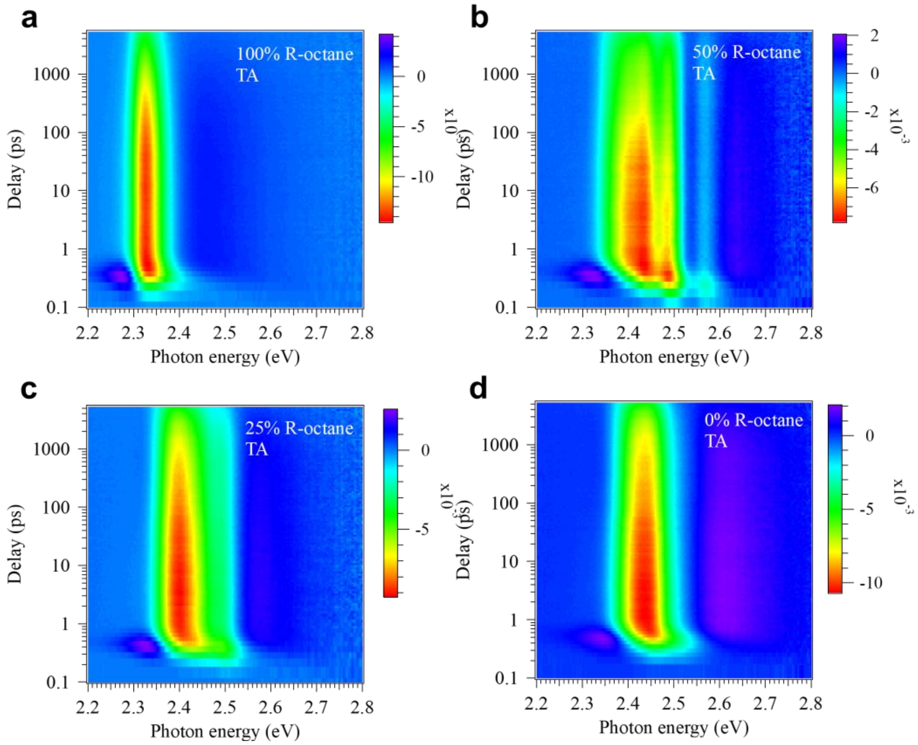

Figure S3. Transient absorption spectrum of $FAPbBr_3$ NCs with different (R)-2-octylamine concentration *x*; a) *x* = 100%, b) *x* = 50%, c) *x* = 25% and d) *x* = 0%. Here, laser with 3.1eV excitation energy (above the bandgap of NCs) was used for the measurement.

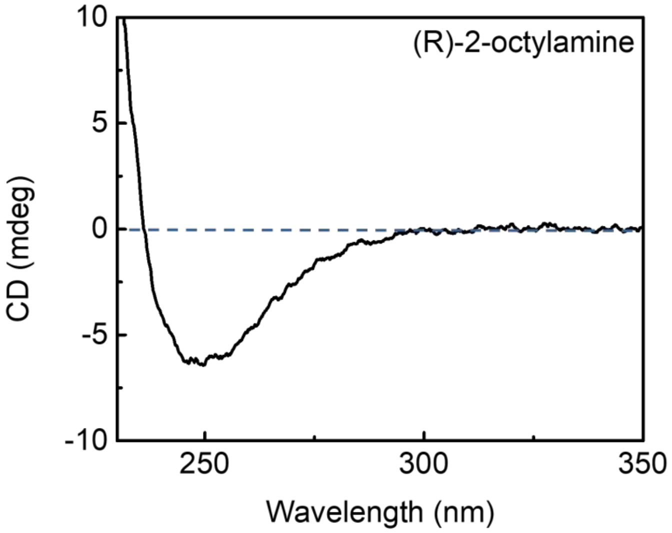

Figure S4. Circular dichroism spectra of (R)-2-octylamine.

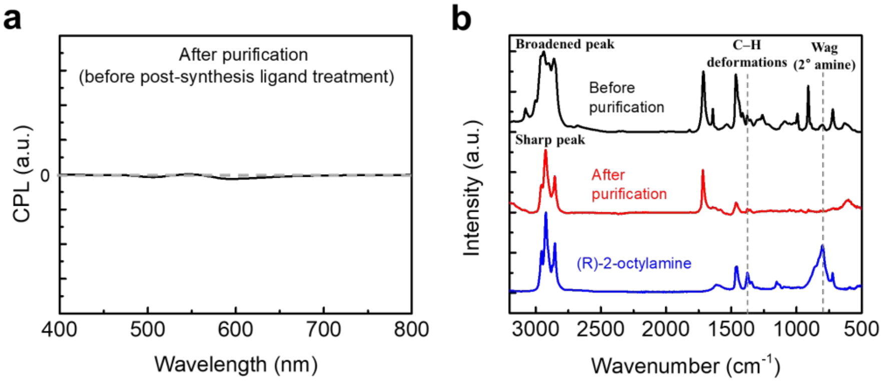

Figure S5. a) CPL of purified $FAPbBr_3$ NCs, b) FT-IR spectrum of (R)-2-octylamine and as-synthesized (before-purified) and purified $FAPbBr_3$ NCs. Here, we tested $FAPbBr_3$ NCs with (R)-2-octylamine concentration x = 50%.

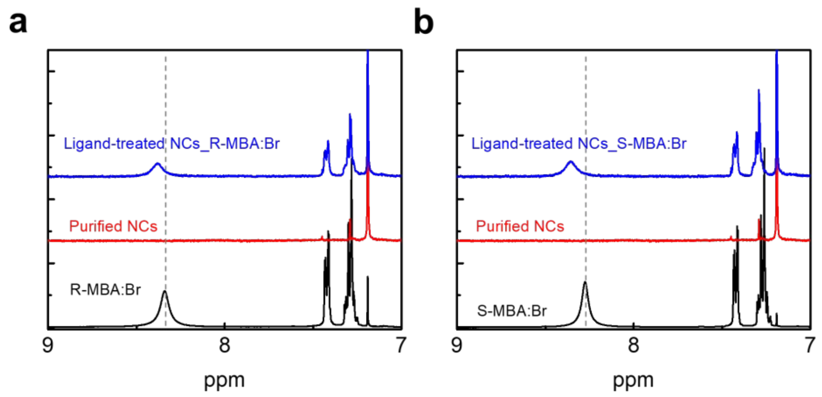

Figure S6. Magnified ^{1}H NMR spectrum (9-7 ppm deg) of a) R-MBA:Br, purified NCs and ligand-treated NCs with R-MBA:Br and b) S-MBA:Br, purified NCs and ligand-treated NCs with S-MBA:Br.

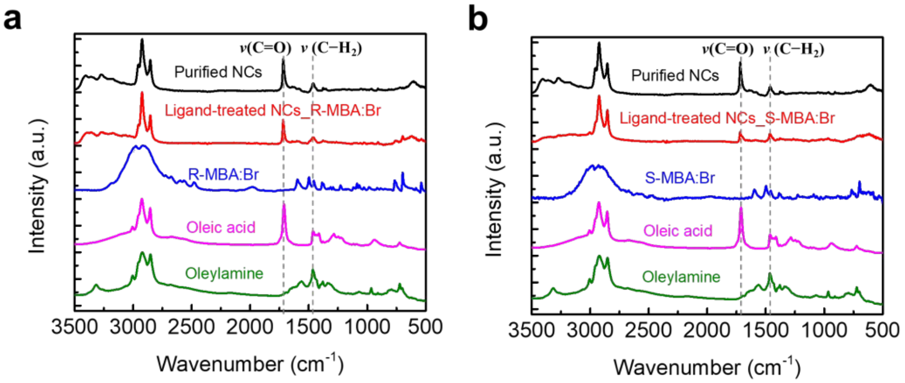

Figure S7. FT-IR spectrum of a) oleic acid, oleylamine, R-MBA:Br, purified NCs and ligand-treated $FAPbBr_3$ NCs with R-MBA:Br and b) oleic acid, oleylamine, S-MBA:Br, purified NCs and ligand-treated $FAPbBr_3$ NCs with S-MBA:Br.

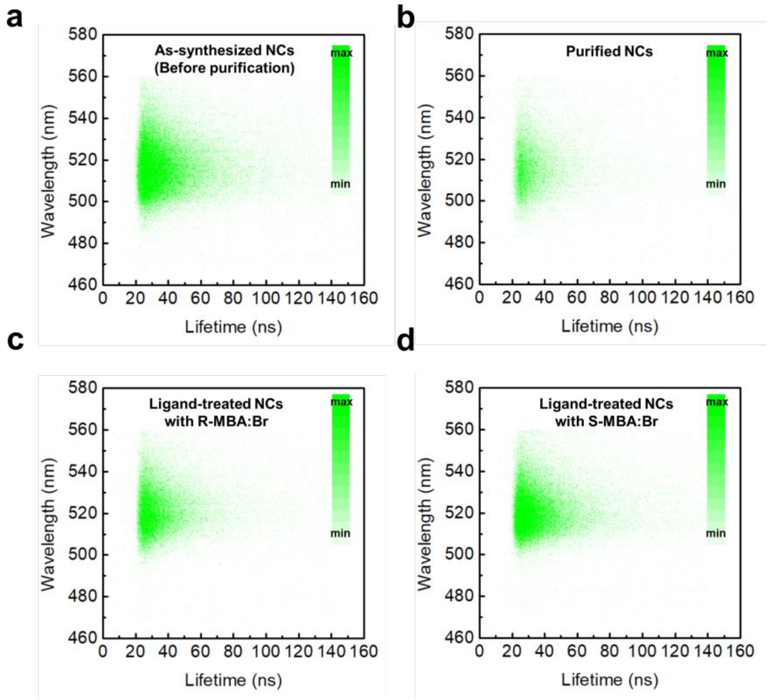

Figure S8. 2-Dimensional mapping of the PL lifetime of a) as-synthesized NCs, b) purified NCs, and ligand-treated NCs with c) R-MBA:Br and d) S-MBA:Br.

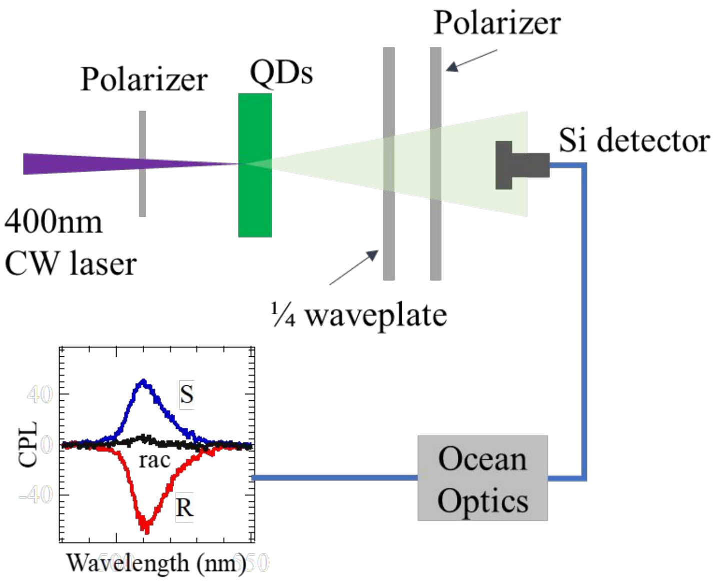

Figure S9. Schematic illustration of CPL measurement system.
